# Association of Proton Pump Inhibitor (PPI) Use with Energy Intake, Physical Activity, and Weight Gain

**DOI:** 10.3390/nu7105416

**Published:** 2015-10-19

**Authors:** Jennifer L. Czwornog, Gregory L. Austin

**Affiliations:** Division of Gastroenterology and Hepatology, University of Colorado Anschutz Medical Campus 12631 E. 17th Ave., Room 7619, Aurora, CO 80045, USA; gregory.austin@ucdenver.edu

**Keywords:** proton pump inhibitor, weight, energy intake, physical activity

## Abstract

Studies suggest proton pump inhibitor (PPI) use impacts body weight regulation, though the effect of PPIs on energy intake, energy extraction, and energy expenditure is unknown. We used data on 3073 eligible adults from the National Health and Nutrition Examination Survey (NHANES). Medication use, energy intake, diet composition, and physical activity were extracted from NHANES. Multivariate regression models included confounding variables. Daily energy intake was similar between PPI users and non-users (*p* = 0.41). Diet composition was similar between the two groups, except that PPI users consumed a slightly greater proportion of calories from fat (34.5% *vs.* 33.2%; *p* = 0.02). PPI users rated themselves as being as physically active as their age/gender-matched peers and reported similar frequencies of walking or biking. However, PPI users were less likely to have participated in muscle-strengthening activities (OR: 0.53; 95% CI: 0.30–0.95). PPI users reported similar sedentary behaviors to non-users. Male PPI users had an increase in weight (of 1.52 ± 0.59 kg; *p* = 0.021) over the previous year compared to non-users, while female PPI users had a non-significant increase in weight. The potential mechanisms for PPI-associated weight gain are unclear as we did not find evidence for significant differences in energy intake or markers of energy expenditure.

## 1. Introduction

More than one third of US adults are obese [1]. Gastroesophageal reflux disease (GERD) is strongly associated with obesity and the prevalence of GERD in the US is estimated to be between 18.1% and 27.8% [2,3]. Patients with GERD often use acid suppressing medications, the most potent of which are proton pump inhibitors (PPI). However, PPI use is costly and has been associated with a number of potential risks [4].

Recently, there has been data to suggest that long-term PPI use leads to weight gain [5]. Acid suppression therapy has also been associated with suboptimal weight loss in patients who underwent laparoscopic Roux-en-Y gastric bypass (LRYGB) [6]. PPI use post-LRYGB may impair weight loss by modification of the gut microbiome and therefore influence energy extraction [7]. However, the extent of weight gain or impaired weight loss associated with PPI use and the potential causes are still largely unknown at this time. Obese patients with GERD are often advised to lose weight, but there is little data on energy intake, diet composition, or physical activity patterns of PPI users compared to PPI non-users.

Given that PPIs may cause weight gain in a population of patients who are more likely to be obese, it is important to understand whether this weight gain is related to energy intake, energy extraction, or energy expenditure. The aims of this cross-sectional study were to use data from the National Health and Nutrition Examination Survey (NHANES) to investigate whether differences in energy intake, diet composition, or physical activity patterns exist for PPI users compared to non-users and whether PPI use is associated with changes in body weight. We hypothesized that PPI use would be associated with increased energy intake, decreased physical activity, and weight gain.

## 2. Methods

### 2.1. Population

Our data are from the NHANES, a complex multistage probability sample of the United States civilian, non-institutionalized population designed to address the health and nutrition status of US adults and children [8]. We used data on 4381 adults aged 20–74 years of age in the 2005–2006 NHANES to determine demographic data, medication use, diet composition, weights, and physical activity levels. There were 3073 individuals who met inclusion criteria and were included in the final analysis. Interviews were used to collect demographic data. Height and weight were measured by standard protocols with calibrated equipment, and BMI was calculated as kg/m^2^. Underweight individuals (BMI < 18.5 kg/m^2^; *n* = 91) and pregnant women (*n* = 313) were excluded. To focus on those not exerting cognitive restraint on food intake, we excluded individuals (*n* = 657) who answered “yes” to the question “Are you currently on any kind of diet, either to lose weight or for some other health-related reason?” All participants provided informed consent and the NHANES study protocol was approved by the National Center for Health Statistic Research Ethics Review Board.

### 2.2. Medication Use

The Dietary Supplements and Prescription Medication Section of the Sample Person Questionnaire collects information on the use of dietary supplements, non-prescription antacid medication use, and prescription medication use. Personal interviews, using the Computer-Assisted Personal Interviewing (CAPI) system, were conducted in the home to collect data on current medication use [8]. All medications and dosages were recorded. Each drug was recorded and eventually entered into a 3-level nested category system that assigns a therapeutic classification to each drug and each ingredient of a drug.

### 2.3. Energy Intake

All participants were asked to complete two 24-h dietary recall interviews, including both weekdays and weekends. All food items and quantities consumed by each participant from midnight to midnight on the day preceding the interviews were recorded. The initial interview was conducted in person. The second interview was conducted by phone 3–10 days later, although not on the same day of the week as the in-person interview. The dietary recalls used the Automated Multiple Pass Method, which is designed to increase the efficiency and accuracy of the 24-h recall by including a thorough compilation of standardized food-specific questions and possible responses [8]. Participants were given a set of measuring guides to help in reporting food amounts during both interviews, as well as a food market booklet to assist in reporting food amounts during the phone interview. The data were used to calculate total energy intake (kcal/day) and the proportion of calories from sugar, non-sugar carbohydrates, saturated fatty acids (SFAs), polyunsaturated fatty acids (PUFAs), monounsaturated fatty acids (MUFAs), protein, and alcohol with the use of the USDA’s Food and Nutrient Database for Dietary Studies [8].

### 2.4. Physical Activity and Sedentary Behaviors

All participants were asked a series of questions about their physical activity. This included responding to the question, “Compared with most men/women your age, would you say that you are more active, less active, or about the same?” Participants were also asked if they participated in specific physical activities (not in the workplace) in the previous 30 days including walking, bicycling, and muscle strengthening activities. If they answered yes to walking or bicycling, they were asked about the frequency and average time they engaged in these activities. Responses for walking or bicycling were recorded as daily, weekly, or monthly. Subsequently, these responses were converted into the total amount of time respondents engaged in these activities in the past 30 days. Because many responses indicated zero or minimal minutes were spent engaging in these activities, the outcome for the frequency of walking or biking in the past 30 days was dichotomized into those who engaged in at least 150 min in the past 30 days and those who had not. This represented those who exceeded 30 min a week in these activities. The outcome for whether participants had engaged in at least 10 consecutive minutes of moderate or vigorous physical activity in the last 30 days was recorded in NHANES as a dichotomous outcome. Similarly, the outcome for muscle-strengthening activity in the past 30 days was recorded in NHANES as a dichotomous response.

Sedentary behaviors were assessed by the questions, “Over the past 30 days, on average about how many hours per day did you sit and watch TV or videos?” and “Over the past 30 days, on average how many hours per day did you use a computer or play computer games?” The responses to these two questions were recorded in NHANES into the following categories: less than 1 h, 1 h, 2 h, 3 h, 4 h, or 5 or more hours. Because of the non-normal distribution of the responses, including large proportions that were “less than 1 h” for both questions, we made an a priori decision to dichotomize these two outcome variables. The responses for watching TV or videos were dichotomized into those who watched TV for 3 or more hours per day and those who watched less than 3 h per day. The responses for computer use were dichotomized into those who used a computer for 2 or more hours per day and those who used a computer for less than 2 h per day.

### 2.5. One-Year Change in Body Weight

Participants were asked to provide (in pounds) what they weighed one year prior in response to the question “How much did you weigh a year ago?” Self-reported weights were converted to weight in kilograms. The outcome of change in body weight over the previous year was calculated as the difference between current measured weight and self-reported weight one year prior.

### 2.6. Covariates

All analyses were adjusted for age, gender, education, race, BMI category (normal weight, overweight, obese), use of insulin, use of non-insulin diabetic medications, use of lipid-lowering medications, use of antidepressants, and total number of non-psychiatric/non-diabetic medications. The response to the question, “Compared with most men/women your age, would you say that you are more active, less active, or about the same?” was also included as a covariate for energy intake because of its strong association with BMI, several other covariates, and energy intake. BMI categories were defined as normal weight (18.5 < BMI < 25), overweight (25 ≤ BMI < 30), and obese (BMI ≥ 30). Underweight individuals were excluded. Education was dichotomized into those who had attended at least some college and those who had a high school degree or less. We coded race as Mexican-American, non-Hispanic white, and non-Hispanic black.

### 2.7. Data Analysis

Statistical analyses were performed using Stata software (version 10.1; StataCorp, College Station, TX, USA). We used survey commands and applied the appropriate sample weights for the data to account for the unequal probabilities of selection. Means (± SE) for percentages and total calories from sugar, non-sugar carbohydrates, protein, SFAs, PUFAs, MUFAs, and alcohol were calculated. Univariate comparisons by PPI use were performed with linear regression. We used multivariable regression analyses (adjusted for the above covariates) to assess the relationship between PPI use and the primary outcomes: daily energy intake, self-assessment of physical activity compared to age/gender-matched peers (more, less, or the same), walking or biking at least 150 min (in the past 30 days), engaging in at least 10 consecutive minutes of any moderate/vigorous physical activity (in the past 30 days), engaging in any muscle strengthening activities (in the past 30 days), and sedentary behaviors.

## 3. Results

Demographic information for both PPI users and those not taking a PPI are shown in Table 1. PPI users were older (*p* < 0.001) and more likely to be white (*p* < 0.001). PPI users had a higher BMI (*p* < 0.001), were more likely to be obese (45.4% *vs*. 30.6%; *p* = 0.008), and were less likely to be normal weight (*p* < 0.001). PPI users were taking more non-acid suppressing medications (*p* < 0.001) compared to non-users. Of those non-acid suppressing medications, PPI users were more frequently taking antidepressants (*p* = 0.001) and lipid lowering medications (*p* < 0.001).

**Table 1:** Patient demographics by use of a proton pump inhibitor (PPI) from the 2005–2006 NHANES.

	PPI non-user (*n* = 2879)	PPI user (*n* = 194)	*p*-value
Age (± SE), year	42.8 ± 0.6	52.8 ± 0.9	<0.001
Gender (% Women)	47.7	47.7	0.992
Race (% White non-Hispanic)	69.7	83.6	<0.001
Completed some college (%)	57.2	55.7	0.741
BMI (± SE), kg/m^2^	27.9 ± 0.3	30.4 ± 0.5	<0.001
Normal-weight (%)	33.9	15.4	<0.001
Overweight (%)	32.7	36.7	0.324
Obese (%)	30.6	45.4	0.008
Number of non-acid suppression medications (± SE)	1.2 ± 0.1	4.0 ± 0.3	<0.001
Taking an Antidepressant (%)	8.3	24.7	0.001
Taking a lipid-lowering medication (%)	9.0	30.3	<0.001

BMI, Body mass index; PPI, proton pump inhibitor; SE, standard error.

### 3.1. Energy Intake and Diet Composition

**Figure 1:**
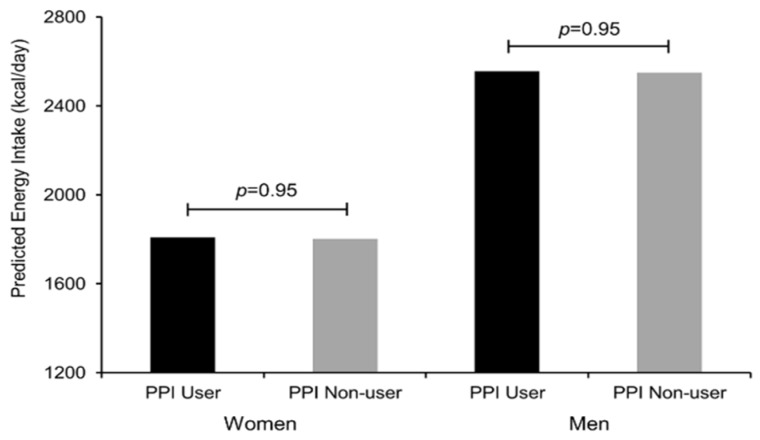
Energy intake (kcal/day) by proton pump inhibitor use for adult men and women in the National Health and Nutrition Examination Survey (NHANES) 2005–2006. Adjusted for use of insulin, non-insulin diabetic medications, use of lipid-lowering medications, antidepressant use, self-assessment of physical activity relative to peers, number of non-psychiatric/non-diabetic drugs, BMI Note: y-axis starts at 1200 kcal/day.

Overall, there was no difference in reported energy intake (kcal/day) among PPI users compared to non-users (*p* = 0.42). Men who were taking a PPI consumed a similar number of calories (mean ± SE) as those who were not taking a PPI: 2556 ± 91 *vs.* 2551 ± 23 kcal/day (*p* = 0.95); the same was true of women PPI users compared to non-users: 1808 ± 78 *vs*. 1802 ± 18 kcal/day (*p* = 0.95) (Figure 1). Subset analyses demonstrated that daily energy intake was also similar among PPI users compared to non-users in the normal weight (*p* = 0.925), overweight (*p* = 0.384), and obese (*p* = 0.846) subgroups. With respect to diet composition, PPI users consumed a slightly higher proportion of calories from fat (34.5% *vs.* 33.2%; *p* = 0.02) and specifically a higher proportion of calories from PUFAs (7.6% *vs.* 7.1%; *p* = 0.003). Consumption of MUFAs was borderline increased among PPI users (12.7% *vs.* 12.2%; *p* = 0.055). PPI users consumed a similar proportion of calories from carbohydrates, both sugar and non-sugar. Also, PPI users and non-users consumed similar proportions of calories from protein and alcohol (Table 2).

**Table 2:** Diet composition by use of a proton pump inhibitor (PPI) from the 2005–2006 NHANES ^1^.

	PPI non-user (*n* = 2879)	PPI user (*n* = 194)	*p*-value ^3^
Macronutrient ^2^ (% energy)			
Carbohydrates (%)	48.1 ± 0.3	48.0 ± 0.7	0.884
Sugar (%)	21.9 ± 0.2	22.1 ± 0.7	0.846
Non-sugar carbohydrates (%)	26.2 ± 0.2	25.9 ± 0.5	0.645
Protein (%)	15.5 ± 0.1	15.0 ± 0.3	0.142
Fat (%)	33.2 ± 0.2	34.5 ± 0.5	0.021
SFAs (%)	11.1 ± 0.1	11.2 ± 0.2	0.565
MUFAs (%)	12.2 ± 0.1	12.7 ± 0.2	0.055
PUFAs (%)	7.1 ± 0.1	7.6 ± 0.2	0.003
Alcohol (%)	3.2 ± 0.2	2.5 ± 0.4	0.139

^1^ Values are means ± SE of percentages; ^2^ SFA: saturated fatty acids; MUFA: monounsaturated fatty acids; PUFA: polyunsaturated fatty acids; ^3^ *p*-values are for the overall F test for each variable.

### 3.2. Physical Activity and Sedentary Behaviors

**Figure 2:**
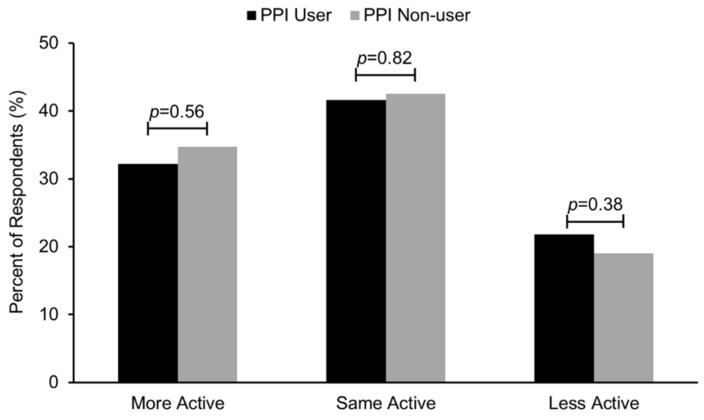
Self-reported physical activity compared to age/gender-matched peers for adult proton pump inhibitor (PPI) users and non-users from the 2005–2006 NHANES. Ajusted for use of insulin, non-insulin diabetic medications, use of lipid-lowering medications, antidepressant use, number of non-psychiatric/non-diabetic drugs, BMI category, education, race/ethnicity, gender, and age.

**Figure 3:**
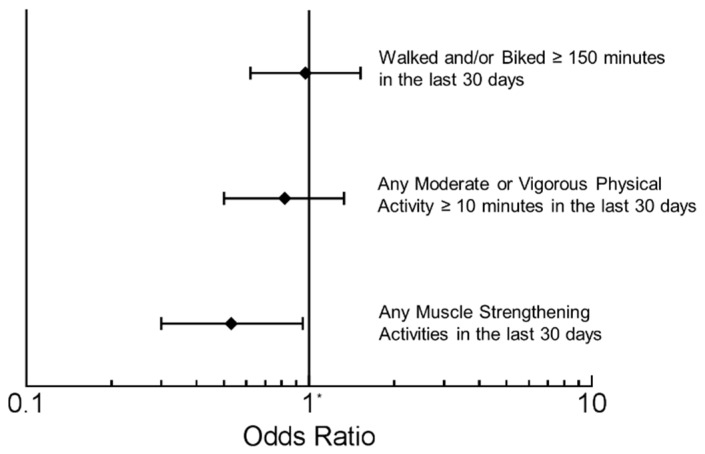
Self-reported physical activities of adults taking proton pump inhibitors (PPIs) in the 2005–2006 NHANES. Adjusted for use of insulin, non-insulin diabetic medications, use of lipid-lowering medications, antidepressant use, number of non-psychiatric/non-diabetic drugs, BMI category, education, race/ethnicity, gender, and age. * Odds Ratio (OR) with 95% CI’s of PPI users compared to non-users.

Study participants were asked if they were more active, less active, or about the same as most men/women their age. PPI users rated themselves as being as physically active as their age/gender-matched peers (Figure 2). The percentage of individuals who walked or biked at least 150 min in the past 30 days was 18.0% (95% CI: 15.6%–20.4%), with PPI users reporting a similar frequency (Figure 3) compared to those not taking a PPI (OR: 0.97; 95% CI: 0.62–1.52). The percentage of individuals who participated in at last ten consecutive minutes of moderate or vigorous physical activity in the prior 30 days was 68.5% (95% CI: 64.8%–72.2%), and PPI users reported a similar frequency compared to non-users (OR: 0.82; 95% CI: 0.50–1.33). The percentage of individuals who participated in muscle-strengthening activities within the previous 30 days was 30.6% (95% CI: 26.4%–34.7%), with PPI users less likely to have engaged in these activities (OR: 0.53; 95% CI: 0.30–0.95). Regarding sedentary behavior, the percentage of individuals who reported spending two or more hours per day using a computer or playing computer games was 16.3% (95% CI: 14.1%–18.4%), with PPI users reporting a similar frequency compared to non-users (OR: 1.02; 95% CI: 0.71–1.48). Similarly, the percentage of individuals who reported spending three or more hours per day watching TV or videos was 36.6% (95% CI: 33.9%–39.3%), with PPI users reporting a similar frequency compared to non-users (OR: 0.90; 95% CI: 0.56–1.46).

### 3.3. One-Year Change in Body Weight

**Figure 4:**
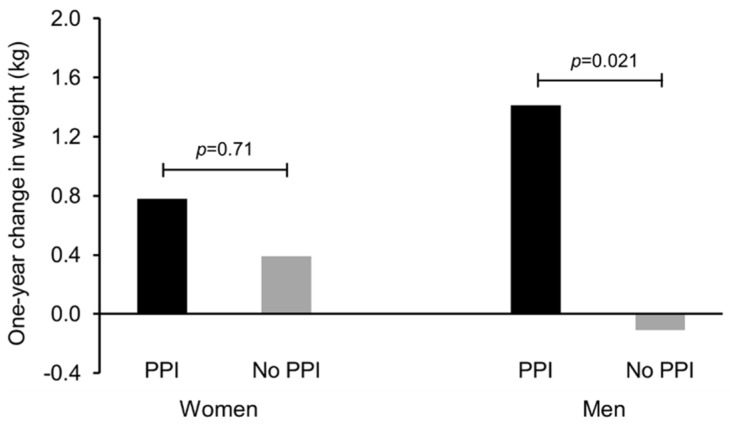
Change in measured body weight compared to self-reported weight one year prior by proton pump inhibitor (PPI) use for men and women in the 2005–2006 NHANES. Adjusted for use of insulin, non-insulin diabetic medications, use of lipid-lowering medications, antidepressant use, number of non-psychiatric/non-diabetic drugs, BMI category, education, race/ethnicity, gender, and age.

The overall mean (± SE) change in body weight for men was a negligible −0.05 ± 0.20 kg. However, the one-year change in body weight was 1.52 ± 0.6 kg greater in male PPI users compared to men who did not use PPIs (*p* = 0.021) (Figure 4). Women overall had a significantly greater increase in body weight (0.66 ± 0.20 kg; *p* = 0.004) compared to all men. The one-year change in body weight was 0.39 ± 1.0 kg greater among women who were PPI users compared to women who did not use PPIs (*p* = 0.71).

## 4. Discussion

PPIs are commonly used by patients with GERD, a disease that is highly associated with obesity. Previous studies reported that PPI use was associated with impaired weight loss following bariatric surgery and weight gain in a non-surgical population [5,6]. Because of the paucity of data on the association of PPI use and weight gain, we aimed to investigate whether PPI use was associated with differences in energy intake, diet composition, physical activity patterns, or changes in body weight using data from the 2005–2006 NHANES. Overall, energy intake was similar between PPI users compared to non-users. With the exception of a mild increase in percent calories from fat, diet composition was similar between PPI users compared to non-users, and this mild increase in percent calories from fat did not lead to an increase in energy intake. With the exception of muscle-strengthening activities, measures of physical activity were also quite similar between PPI users and non-users. However, we found that PPI use, primarily among men, was associated with a significant increase in body weight.

This finding of an increase in body weight, primarily among men, was one of the most interesting findings in this study. While we cannot ensure causality, in part because of the timeframe of medication use and weight recall, our findings are consistent with the previous report that PPI use is associated with significant increases in body weight. In the study by Yoshikawa *et al.*, initiation and long-term PPI use was associated with at least a 5% weight gain in 36% of patients using PPIs compared to only 4% of patients who were not using a PPI over a two-year period of follow-up [5]. However, the cause of the weight gain was not evaluated in that study, and GERD patients taking PPIs were compared with age-matched healthy controls, as opposed to other GERD patients who were similarly symptomatic but who did not receive a PPI. This study is novel in that we investigated potential causes of PPI-associated weight gain and whether measures of energy intake and energy expenditure differed between PPI users and non-users.

Because patients on PPIs might have fewer postprandial symptoms, we had hypothesized that if PPI use was associated with weight gain that it would be the result of increased energy intake. We did not find any evidence that PPI users consume more calories compared to non-users. The small one percentage point increase in percent calories from fat for PPI users compared to non-users did not translate into an increase in overall energy intake. However, because this is a cross-sectional study that assessed energy intake using dietary recall, we cannot exclude that PPI use may lead to increased energy intake. There are limitations to dietary recall as a method of assessing energy intake, as dietary recall may best reflect short-term and not long-term dietary patterns [9]. Individuals often underreport energy intake, and obese individuals are more likely to underreport energy intake. However, because we controlled for being overweight or obese in our models, selective underreporting among PPI users is very unlikely to be the cause of falsely concluding that energy intake is similar between PPI users and non-users. Furthermore, we performed subset analyses within the normal-weight, overweight, and obese subgroups and did not find any association between PPI use and energy intake in these subgroups.

To understand both sides of the energy balance equation, we also assessed measures of physical activity and sedentary behavior. The responses to the question “Compared with most men/women your age, would you say that you are more active, less active, or about the same?” were nearly identical in PPI users compared to non-users. Compared to non-users, PPI users were as likely to have engaged in at least 150 min of walking and/or biking and to have engaged in at least 10 consecutive minutes of moderate or vigorous physical activity in the prior 30 days. The only significant difference between PPI users and non-users was that PPI users were less likely to have engaged in muscle strengthening exercises in the prior 30 days. Studies have shown that women who met muscle-strengthening recommendations had a significantly lower BMI, a lower percentage of body fat, higher muscle strength, and were less likely to be obese [10]. There is also an inverse relationship between muscle strength and excessive abdominal fat [11]. However, the significance in this study of PPI users being less likely to have engaged in muscle strengthening activities is unclear in regards to one-year changes in body weight, as engaging in muscle strengthening exercises (in the prior 30 days) was not associated with one-year changes in body weight in either men or women in this study (data not shown). One study which investigated PPI use in patients with erosive esophagitis with a BMI of >25 found that a hypocaloric diet and aerobic exercise program allowed more patients than those with a standard diet to reduce or stop PPI therapy [12]. We cannot exclude the possibility that some NHANES participants had GERD but had previously lost weight and were able to discontinue their PPI use.

The reasons for the difference associations of PPI use with one-year body weight changes between genders are unclear. Previous studies have reported men gain less weight over a four-year period than women, though this was found in a population which is dissimilar to NHANES [13]. Similarly, women in this study had a mean change in body weight over the previous year that was 0.66 kg greater compared to the mean change in body weight for men. The overall trend of increasing body weight in women might obscure the potential effect of PPIs on weight gain in this group.

One possible explanation for how PPIs may influence weight is through increased energy extraction. PPI use has been associated with an increase in the percent relative abundance of bacteria from the phylum Firmicutes and a decrease in the percent relative abundance of bacteria from the phylum Bacteroidetes, a profile associated with obesity and increased energy harvest from the diet [7,14]. This study did not examine mechanisms for energy extraction, but it is an area that should be further investigated.

We acknowledge some limitations to this study. One limitation is the use of participant recall for diet, physical activity, and weight data. Underreporting is common in 24-h dietary recall studies, and obese individuals may be more likely to underreport energy intake [15,16]. However, as stated above, we both controlled for being overweight and obese and performed subgroup analyses within the normal weight, overweight, and obese participants and did not find an association with PPI use and energy intake. Future studies should use more objective measures of energy intake and energy expenditure (doubly-labeled water and wearable devices) [17,18]. Because of the cross-sectional nature of the study, there may be other unmeasured confounders that might alter the observed associations between PPI use and the outcomes. However, known cofounders associated with the outcomes were included in our analysis.

## 5. Conclusions

PPI use was associated with a significant weight gain in men and a non-significant weight gain in women. Measures of energy intake, physical activity, and sedentary behavior were similar between PPI users and non-users in both men and women. Further investigation to confirm the association of PPI use with weight gain should include more objective measures of energy intake and energy expenditure and should address mechanisms that contribute to this weight gain.
